# *M. avium Complex* Pulmonary Infections: Therapeutic Obstacles and Progress in Drug Development

**DOI:** 10.3390/ph18060891

**Published:** 2025-06-13

**Authors:** Elise Si Ahmed Charrier, Alexandra Dassonville-Klimpt, Claire Andréjak, Pascal Sonnet

**Affiliations:** 1 Agents Infectieux, Résistance et Chimiothérapie, AGIR, Université de Picardie-Jules-Verne, UR 4294, UFR de Pharmacie, 1 Rue des Louvels, CEDEX 1, 80037 Amiens, France; elise.charrier@u-picardie.fr (E.S.A.C.); alexandra.dassonville@u-picardie.fr (A.D.-K.); claire.andrejak@u-picardie.fr (C.A.); 2 Service de Pneumologie, CHU Amiens-Picardie, 80000 Amiens, France

**Keywords:** non-tuberculous mycobacteria, *Mycobacterium avium complex*, anti-MAC compounds, drug development, clinical trials

## Abstract

Worldwide, several million people are infected with mycobacteria such as *Mycobacterium tuberculosis* (*M. tb*) or non-tuberculous mycobacteria (NTM). In 2023, 10.8 million cases and 1.25 million deaths due to *M. tb* were recorded. In Europe and North America, the emergence of NTM is tending to outstrip that of *M. tb*. Among pulmonary NTM, *Mycobacterium avium complex* (MAC) is the most common, accounting for 80% of NTM infections. First-line treatment requires the combination of at least three antibiotics over a long period and with different mechanisms of action to limit cross-resistance. The challenge is to discover more effective new anti-MAC molecules to reduce the duration of treatment and to overcome resistant strains. The aim of this review is to present an overview of the challenges posed by MAC infection such as side effects, reinfections and resistance mechanisms. The latest therapeutic options such as the optimized combination therapy, drug repurposing and the development of new formulations, as well as new anti-MAC compounds currently in (pre)clinical trials will also be discussed.

## 1. Introduction

Mycobacteria belong to the bacteria family and are fine and immobile bacilli (1 to 10 µm long and 0.2 to 0.6 µm wide) whose branched filaments resemble fungi, hence the prefix “myco”. Mycobacteria are divided into three categories: *Mycobacterium leprae*, *Mycobacterium tuberculosis* (*M. tb*) complex and non-tuberculous mycobacteria (NTM). NTM are ubiquitous, i.e., present in the environment, particularly in water and soil, and are opportunistic. Responsible for localized or disseminated infections, they affect the skin, soft tissues, etc., and mainly the lungs (65% of infections). They occurred in immunocompromised people and/or, those with a chronic respiratory disease (cystic fibrosis (CF), non-CF bronchiectasis and chronic obstructive pulmonary disease (COPD)).

However, even if *M. tb* is the most known of mycobacteria, as responsible for 10.8 million infections and 1.25 million deaths worldwide in 2023, NTM infections are emerging. In some regions of the world, they tend to exceed those caused by *M. tb* [1,2]. This trend is probably linked to an increase in the number of patients at risk, with both a rise in the incidence of chronic respiratory pathologies and an aging population in certain regions of the world. To date, 190 NTM species have been described and classified into two groups based on their growth rate: rapidly growing mycobacteria (RGM) and slowly growing mycobacteria (SGM) [3]. Representative RGM comprises *M. abscessus* complex, *M. fortuitum* and *M. chelonae* while examples of SGM include, for example, the *M. avium* complex (MAC) and *M. xenopi*. Among the 190 species identified, six are recognized as pulmonary pathogens: *M. abscessus*, MAC, *M. xenopi*, *M. kansasii*, *M. malmoense* and *M. szulgai* [4].

NTM-related lung diseases are very difficult to diagnose; current treatments are not sufficiently effective and antibiotic resistance is increasing. Such issues underscore the urgent need to develop new molecules that are safer, selectively target NTM and ideally operate via a novel mechanism of action. In this review, we focus on MAC because they are the most prevalent NTM and responsible for almost 80% of lung infections in populations worldwide [3]. The guidelines-based therapy (GBT) recommended by the American Thoracic Society (ATS)/Infectious Diseases Society of America (IDSA) criteria (2020) to treat MAC lung disease is a combination of three antibiotics: a macrolide such as **clarithromycin** (**CLR**) or **azithromycin** (**AZI**), a rifamycin like **rifampicin (RIF)** or **rifabutin** (**RIB**) and **ethambutol** (**EMB**) [5]. The duration of treatment is at least twelve months but its effectiveness is moderate, with an estimated success rate of 52–66% partly due to the emergence of macrolide resistance [6]. In addition, this treatment induces numerous undesirable side effects. Therefore, novel anti-MAC drugs are urgently needed to improve outcomes in MAC lung infections. This review presents an overview of MAC infections, available regimens and various therapeutic obstacles, such as resistance mechanisms. We also discuss the latest therapeutic options, namely the optimization of current treatments, the development of new formulations, the repurposing of existing drugs and the discovery of new compounds in (pre)clinical development. For each of these approaches, different families of compounds will be presented, with their in vitro and in vivo biological properties, pharmacokinetic (PK) parameters, mode of action, structure-activity relationships (SAR) and results from clinical trials.

## 2. Overview of MAC Infection

### 2.1. Subspecies of MAC

Three major species of MAC exist such as *M. intracellulare, M. chimaera* (sometimes presented as *M. intracellulare-chimaera* as they are very close) and *M. avium*. In the 1940s, *M. intracellulare* ATCC 13950 was isolated from a child who died of disseminated disease associated with genetic immunological deficiency [7]. *M. intracellulare* subspecies is more prevalent and the number of colony-forming units (CFUs) is higher than that of *M. avium* in biofilms. However, *M. avium* is more frequent in clinical isolates [8]. It includes four subspecies: *M. avium* subsp. *avium*, *M. avium* subsp. *paratuberculosis*, *M. avium* subsp. *silvaticum* and *M. avium* subsp. *hominissuis* [4]. *M. avium* subsp. *avium* ATCC 25291 was first isolated in 1890 from a chicken and, in 1930, identified in humans suffering from acquired immunodeficiency syndrome, lymphadenitis and chronic lung disease such as CF. *M. avium* subsp. *paratuberculosis* ATCC 19698, was isolated from the cow. This subspecies was later subdivided into two groups: bovine and ovine. *M. avium* subsp. *silvaticum* ATCC 48898 was isolated from the liver and spleen of a wood pigeon. *M. avium* subsp. *hominissuis* isolated from the pigs [9].

### 2.2. Pathogenesis

#### 2.2.1. Pathophysiology

MAC infections typically occur via the respiratory route, with the pulmonary alveoli serving as the primary site of infection. During sleep, patients may inadvertently swallow pharyngeal secretions containing MAC [10]. Due to their resistance to the acidic environment of the stomach, these bacteria can survive gastrointestinal transit and penetrate the intestinal epithelial mucosa. They subsequently translocate across the mucosa by entering enterocytes [11]. Another mechanism for escaping epithelial cells is to interact with fibronectin-attachment protein to bind the integrin receptors located in the cell membrane of mucosal cells [12]. This entry into mucosal cells is associated with a suppression of IL-8 and RANTES production. As a result, MAC can establish an infectious niche prior to the host immune response. If the immune system is compromised, it can survive in the lymph nodes. MAC is also able to invade type II alveolar epithelial cells and replicate them within these cells.

Once in the host, MACs enter via various receptors (complement receptor CR3, mannose receptors, etc.) and live in mononuclear phagocytes such as monocytes and macrophages [12]. Phagosomes fuse with vacuoles inside the macrophage cytoplasm. As MAC is an intracellular pathogen, it survives and proliferates within vacuoles.

Additionally, the presence of glycopeptidolipids in the MAC cell membrane alters macrophage function, preventing fusion of the phagosome with the liposome and limiting exposure to hydrolytic enzymes. Following apoptosis, MAC leaves the first macrophage and invades a second uninfected macrophage using scavengers and transferrin receptors. This mechanism is responsible for MAC dissemination [12].

Macrophages play an important role in host defense against MAC. They activate NK cells, stimulate cytokine production (IL-12, TNF-α, IFN-γ and granulocyte macrophage colony-stimulating factor) and T lymphocytes proliferate [11].

#### 2.2.2. Risk Factors and Comorbidities

Risk factors associated with MAC infections include environmental and host factors. As mentioned above, MACs are ubiquitous, particularly in water and soil. Colonization and persistence of MAC in water are due to biofilm formation [13], high temperature resistance, disinfectants resistance and growth at low oxygen concentration [14,15]. Environmental exposure is, therefore, a major risk factor for human infection.

**Figure 1:**
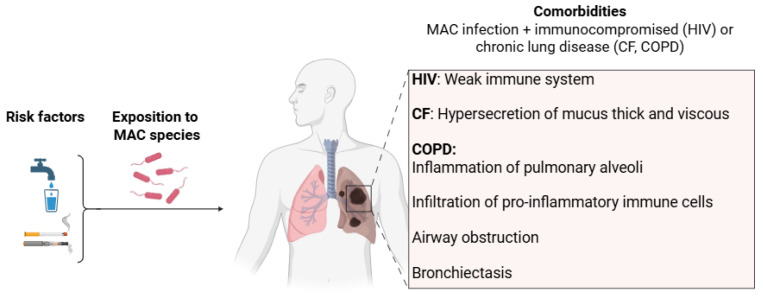
Risk factors and comorbidities associated with MAC infections.

Concerning comorbidities, structural pulmonary diseases such as nodular bronchiectasis and COPD are the main risk factors. Bronchiectasis, whether or not of CF origin, manifests itself as abnormal bronchial enlargement caused by persistent inflammation, leading to reduced vibratory cilia function and increased mucus production (Figure 1). COPD is characterized by systemic inflammation of the pulmonary alveoli, infiltration of pro-inflammatory immune cells such as eosinophils, T lymphocytes and macrophages, airway obstruction and hypersecretion of mucus [16]. The warm and humid environment of the viscous mucus is conducive to the growth of mycobacteria and thus to the onset of infection. In addition, it has been shown that inhaled corticoids prescribed for bronchiectasis and COPD significantly increase the risk of NTM infections [17].

### 2.3. Diagnosis Criteria

The clinical symptoms are variable and nonspecific such as cough, sputum, fever, dyspnea, weight loss, fatigue, etc. To eliminate the most likely diseases with regard to non-specific symptoms, the diagnosis takes account of radiological and microbiological manifestations. Moreover, it is necessary to exclude all others diagnoses which could be responsible for clinical and radiological symptoms.

**Figure 2:**
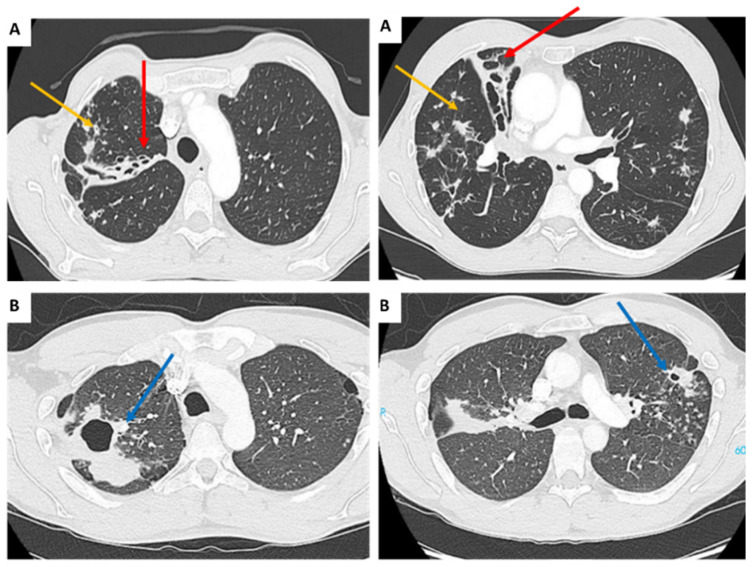
Computed tomography scan findings in two MAC-infected patients with (**A**) nodular bronchiectasis pattern with bronchial wall thickening (red arrows), centrilobular nodules and mucus plugging (yellow arrows) and (**B**) cavitary pattern with multiple cavitating lesions (blue arrow).

The radiological diagnosis distinguishes between two different diseases: nodular bronchiectatic disease (ND) and cavitary disease (CD). These two main patterns are diagnosed by high-resolution computed tomography scan (Figure 2) [18]. The nodular bronchiectasis pattern occurs preferentially in women who are taller and thinner than average. It is associated with lesions, dilatation and destruction of the bronchi, as well as a mucus plug, particularly in the middle lobe, which is the most difficult to drain. Management of bronchiectasis includes treatment to limit airway obstruction, such as bronchodilators, and improve airway clearance, such as mucoactive agents, in combination with respiratory physiotherapy. Nodules eventually develop into cavities. Cavitation is most common in COPD patients. It is characterized by the air-containing spaces in all lobes, with a preference for the apical and posterior segments. The thin, smooth bronchial wall may also become thick and irregular [18,19].

Microbiological criteria are also essential for the diagnosis of NTM infections. According to the ATS/IDSA criteria (2020), positive cultures must be observed from (i) at least two expectorated sputum samples separated by at least 7 days or, (ii) at least one bronchial wash or, (iii) transbronchial or other lung biopsy and positive culture for NTM with mycobacterial histopathologic features [2,5]. Microbiologic criteria are verified using identification markers specific to MAC.

## 3. Available Drugs for MAC Infections and Challenges

### 3.1. Available Medications for Treating MAC Infections

**Figure 3:**
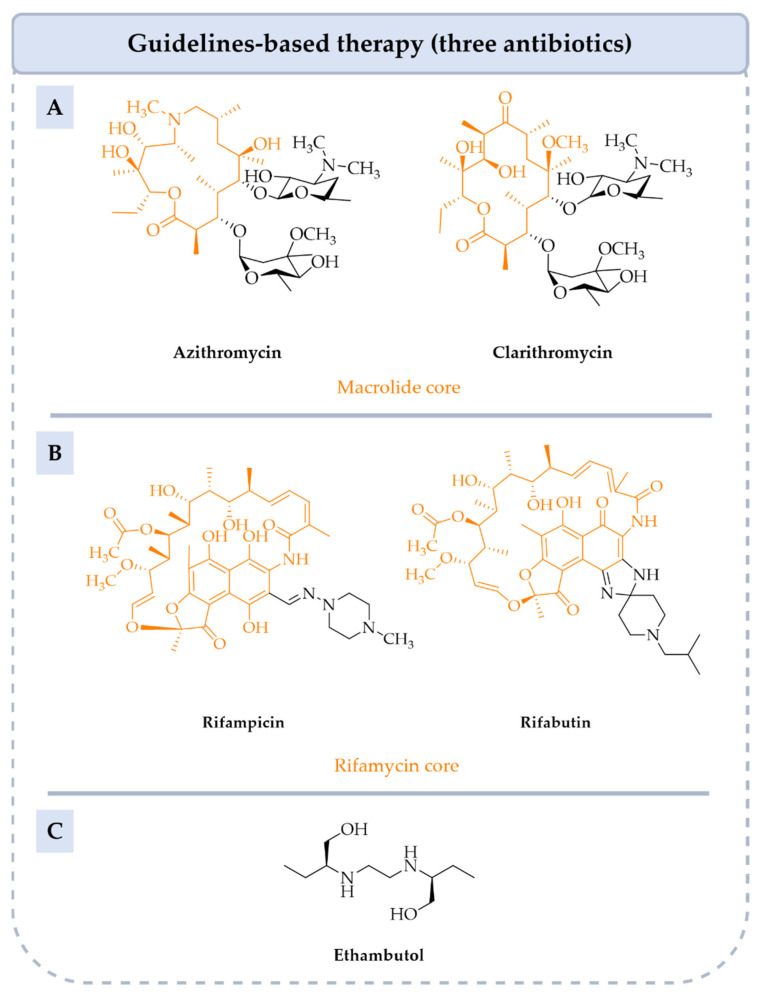
Antimycobacterial drugs of GBT used to treat MAC infections. (**A**): Structure of **AZI** and **CLR** (macrolide core in orange). (**B**): Structure of **RIF** and **RIB** (rifamycin core in orange). (**C**): Structure of **EMB**.

Current drugs prescribed to treat NTM infections were originally developed to eradicate *M. tb*. The GBT recommended by the ATS to treat nodular bronchiectatic MAC lung disease consists of a combination of three antibiotics: macrolides such as **CLR** or **AZI**, rifamycins like **RIF** or **RIB** and **EMB** (Figure 3). Medical guidelines to treat ND are different between Europe and North America (Table 1). GBT can be prescribed three times a week or daily to prevent the emergence of resistant strains. In the case of CD, GBT is prescribed with the addition of **amikacin** IV (**AMK**) three times per week or daily according to recommendations [2,5,20]. In both Europe and North America, in cases of refractory disease (RD), i.e., lack of response after six months of GBT, **AMK liposome inhalation suspension** (**ALIS**) or **AMK IV** or **streptomycin** can be added to GBT [2,20]. However, it is important to note that the use of **streptomycin** has declined considerably due to the availability of newer, safer and more effective drugs and that it is no longer available in many countries, including France. Other antibiotics may be included such as **clofazimine** (**CFZ**) as a replacement for **RIF** [5].

Treatment duration for MAC-infected patients should be at least twelve months after culture conversion. Some patients consider stopping the treatment early due to adverse effects, but this increases the risk of diseases relapsing with the original strain. In patients who complete the treatment, reinfection is also common, especially in those with bronchiectasis, often due to a new strain of MAC [2]. Exposure to mycobacteria persists, given their ubiquitous presence in the environment, as do risk factors, notably chronic respiratory pathologies such as bronchiectasis or COPD. Thus, a patient who has had an NTM infection is at high risk of re-infection. Actions to limit exposure can be proposed. Diel et al. estimated a treatment efficacy of 52% for MAC-infected patients treated previously with a combination of drugs including a macrolide [6]. For macrolide-susceptible patients not previously treated, efficacy was 66% [6]. The MAC treatment regimen leads to more or less serious adverse effects, such as hepatotoxicity, nausea and vomiting (**CLR**/**AZI**), orange coloration of secretions (**RIF**), ocular disorders (**EMB**) and systemic, renal or ototoxicity (**AMK**, **streptomycin**) [2]. In addition, **CFZ** causes an orange pigmentation of the skin, due to its riminophenazine core, and abdominal pains [21]. Drug interactions are common, especially with **RIF** which is a strong enzyme inducer [22]. **CLR** also interacts with many drugs via cytochrome P450. In particular, it increases the toxicity of **RIB**.

To sum up, treatments are long, moderately effective and have numerous side effects. Furthermore, MAC strains are increasingly resistant to current treatments, especially macrolides.

### 3.2. Resistance Mechanisms

#### 3.2.1. Unique Features of the NTM Cell Envelope as Sources of Intrinsic Antibiotic Resistance

**Figure 4:**
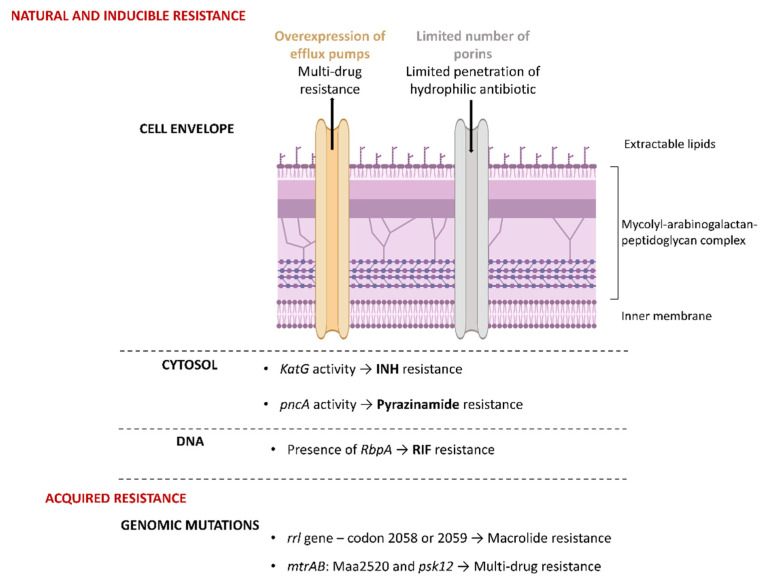
Mechanisms of natural, inducible and acquired antibiotic resistance in *M. avium complex*.

Mycobacteria have exceptionally low-permeability cell walls making them resistant to therapeutic agents, particularly hydrophilic antibiotics, and contributing to their pathogenicity. Their thick, waxy outer layer limits drug permeability, restricts drug penetration and shields them from desiccation, disinfectants and host immune defenses. The cell envelope of mycobacteria comprises a capsule, different extractable lipids, a mycolyl-arabinogalactan-peptidoglycan complex composed of successive layers of covalently bonded mycolic acids, arabinogalactan, peptidoglycan and an inner membrane (Figure 4). Mycolic acids are fatty acids with a long chain of 70 to 90 carbons and represent up to 60% of the weight of the mycobacterial cell wall and around 40% of the mycolyl-arabinogalactan-peptidoglycan complex. In addition, mycolic acids are associated with glycolipids, particularly trehalose glycolipids (e.g., trehalose dimycolate). These extractable cell wall lipids differ in location, nature and proportion between NTM species [23,24]. Proper synthesis and export of these major cell envelope constituents via transporters such as MmpL3 is essential for viability. Porins also play an important role in transporting molecules across the mycobacterial cell membrane. In mycobacteria, porins proteins are less abundant and less efficient than in other bacteria, which slows down the diffusion of hydrophilic drugs.

Thus, the abundance of mycolic acids and the limited number of porins contribute to the highly hydrophobic cell wall and their intrinsic resistance, particularly against many hydrophilic antibiotics.

#### 3.2.2. Other Resistance Mechanisms

Unlike *M. tb*, many NTM species are naturally resistant to drugs like **isoniazid** (**INH**) and **pyrazinamide** due to structural differences within the two enzymes involved, **katG** (catalase-peroxidase) and pncA (pyrazinamidase), respectively [25,26]. **RIF** exerts its antibacterial effect by binding to the β-subunit (*RpoB*) of RNA polymerase, thereby preventing RNA synthesis. Resistance to **RIF** in mycobacteria, and particularly in *M. tb*, MAC and *M. abscessus*, could be explained by mutations in the *RpoB* gene or by an inducible mechanism due to RNA polymerase-binding protein A (*RbpA*) [27]. *RbpA* is an RNA polymerase-associated protein that binds to the RNA polymerase complex near the *RpoB* subunit. When *RbpA* is attached to RNA polymerase, it alters the conformation of the **RIF**-binding pocket, reducing the ability of **RIF** to bind and inhibit transcription [28].

Macrolides, rifamycins, aminoglycosides and other antibiotics can be substrates of efflux pump systems (e.g., ABC transporters, MmpL proteins) which are widely present in NTM and actively expel antibiotics, thereby reducing their intra-cellular concentration.

NTM macrolide resistance is particularly problematic because this antibiotic class plays a crucial role in treating NTM disease. The high level of macrolide resistance in MAC can be partly explained by acquired genomic mutations. Macrolides bind to the 23S rRNA at the peptidyl transferase center, inhibiting bacterial protein synthesis. Mutations (e.g., A2058G, A2059G) in the macrolide-binding site of the 23S rRNA, encoded by the *rrl* gene, are associated with high-level macrolide resistance in MAC and *M. abscessus*. The development of macrolide resistance can be prevented by using multi-drug regimens that include **RIF** and **EMB** [27,29]. A number of genes and systems are also involved in multi-drug resistance. The main system in the *Mycobacterium* genus is *mtrAB* and the two main genes in MAC are Maa2520 and *pks12* (or Maa1979) [30]. The Maa2520 gene may code for an exported protein, while the *pks12* gene code for a polyketide synthase necessary for the synthesis of dimycocerosyl phthiocerol, an important cell wall constituent. As with *M. abscessus*, MAC can form smooth transparent or smooth opaque colonies (rough colonies of MAC are rare) [31]. The smooth, opaque colony type is more sensitive to **CLR**, for example. This sensitivity is regulated by *mtrAB*. The *pks12* gene is highly conserved in actinomycetes but its role in cell wall maintenance is more or less important depending on NTM species. For example, it plays a less important role in *M. tb* than in MAC.

In vivo studies have also shown that the persistence of MAC is due to its ability to establish biofilms [32].

### 3.3. Challenges

There are many challenges to the development of new molecules against MAC. As mentioned above, mycobacteria are highly unusual bacteria, with a lipid-rich wall and numerous resistance mechanisms that render inactive many antibiotics used to treat Gram-positive or Gram-negative bacterial infections. Furthermore, treating an NTM infection requires the use of several antibiotics in combination, to limit the risk of resistance emergence. It is therefore essential to ensure that there is no antagonism between antibiotics (hence the importance of assessing synergies using checkerboard methods). Moreover, combining antibiotics can increase the potential toxicity of the different molecules used. We must therefore always ensure that there is no potentiation of toxicity, but also no antagonism with key molecules;, i.e., macrolides for MAC. This is all the more important given the need to consider both short-term and cumulative toxicity, given the long duration of the treatment.

## 4. Current and Future Drug Targets for the Treatment of MAC Lung Infections

**Figure 5:**
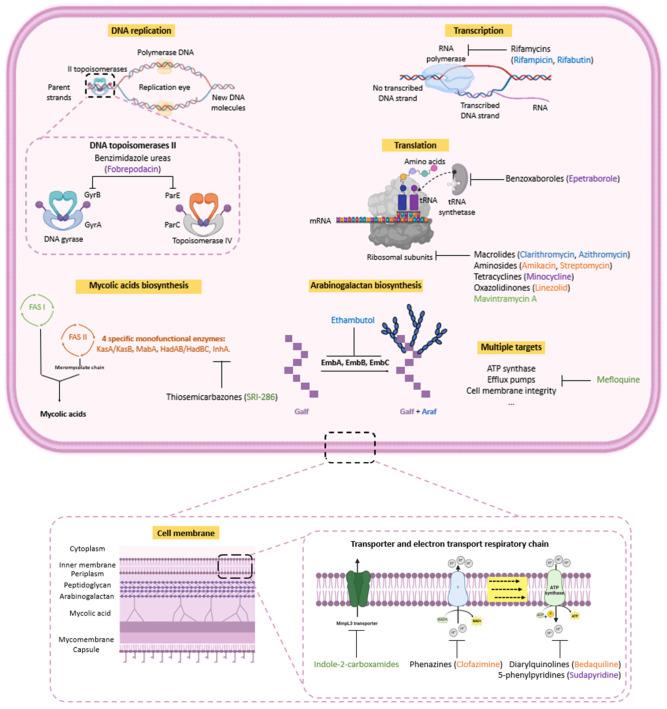
Targets and mechanisms of action of current in first-line treatment (blue) and second-line treatment (orange) antimycobacterial drugs. New compounds in preclinical trials and clinical trials are shown in green and purple, respectively.

Antimycobacterial drugs of GBT target essential enzymes involved in RNA-to-protein translation such as macrolides, in DNA transcription such as rifamycins and in membrane wall formation such as **EMB** (Figure 5). More precisely, **CLR** and **AZI**, bind to the 50S ribosomal subunits, inhibiting protein synthesis by blocking the ribosome exit tunnel, thus preventing the newly synthesized peptide chain from being released [33,34]. This blockage interferes with the elongation process, interrupting bacterial protein production. Rifamycins such as **RIF** and **RIB** target the β-subunit of the bacterial RNA polymerase, preventing the transcription of bacterial DNA into messenger RNA (mRNA) required for protein production [35]. Thus, bacterial protein synthesis is disrupted, leading to bacterial cell death or inhibition of bacterial growth. **EMB** inhibits the activity of three arabinosyl transferases (EmbA, EmbB and EmbC), responsible for transferring arabinose to the growing arabinogalactane polymer, a crucial component of the mycobacterial cell wall [36,37].

Among the antimycobacterial drugs used to treat severe pulmonary infection, aminoglycosides like **AMK** bind to the 30S subunit of the mycobacterial ribosome. This binding interferes with the initiation of protein synthesis, causing protein translation errors [38]. Phenazines such as **CFZ** target NADH-quinone oxidoreductase II, an enzyme involved in the electron transport chain, preventing bacteria from generating energy through cell respiration. Additionally, **CFZ** increases the activity of the phospholipase A_2_, leading to the release of lysophospholipids [39], contributing to bacterial cell damage by promoting oxidative stress.

Some molecules used in antituberculosis therapy have been prescribed to patients who cannot tolerate standard treatments or when no other alternatives are available (only case reports). This is the case with **bedaquiline** (**BQ**) which targets mycobacterial ATP synthase, another enzyme involved in the electron transport chain, thus depriving the bacteria of the energy they need to survive [40]. **Sudapyridine**, a new diarylquinoline, is currently undergoing clinical studies on *M. tb* and a preclinical phase on MAC. **Linezolid**, an oxazolidinone antibiotic, used to treat Gram-positive infections and as a second-line treatment for multidrug-resistant tuberculosis, binds to the 23S rRNA of the bacterial ribosome inhibiting protein synthesis [41].

Other repurposed antibacterial drugs, such as **minocycline**, are currently the subject of case reports to treat MAC infections. **Minocycline**, a broad-spectrum tetracycline antibiotic, binds to the **30S subunit** of the bacterial ribosome, preventing the attachment of aminoacyl-tRNA. This binding inhibits the addition of amino acids to the growing polypeptide chain during protein synthesis.

Few anti-MAC compounds with a novel mode of action are in clinical trials. These include **fobrepodacin**, **epetraborole** (Figure 4, molecules colored in purple). **Fobrepodacin**, a benzimidazole urea, targets the ATP-binding sites of the GyrB subunit of DNA gyrase and the ParE subunit of topoisomerase IV, two enzymes involved in DNA replication and supercoiling in bacteria [42]. **Epetraborole**, a benzoxaborole, targets the leucyl-tRNA synthetase, an enzyme that plays a crucial role in **protein synthesis** by attaching the amino acid **leucine** to its corresponding **tRNA (transfer RNA).** This **aminoacylation** process is essential for translating genetic code into functional proteins.

Lastly, four compounds or families of compounds are in preclinical studies: **SRI-286**, indole-2-carboxamide family, **mavintramycin A** and **mefloquine** (**MQ**) (Figure 4, molecules colored in green).

**SRI-286**, a thiosemicarbazone, is known to inhibit InhA, a specific enzyme of Fatty Acid System II (FAS-II), involved in the biosynthesis of bacterial fatty acids such as mycolic acids. Among the chemical families in preclinical studies, indole-2-carboxamides, inhibit an essential transporter efflux pump MmpL3 which is responsible for exporting trehalose monomycolates, which are precursors for mycolic acid attachment to the cell envelope. **Mavintramycin A** is a naturally occurring aminoglycoside antibiotic, acting as a congener of **AMK**. The latest compound is **MQ**, an antimalarial used for over 40 years. It has been repositioned against NTM. Studies describe its ability to inhibit ATP synthase in certain bacteria such as Streptococcus pneumonia [43]. Other studies highlight its use as an adjuvant in combination with conventional antibacterial and anti-*M. tb* agents [44,45,46]. In fact, **MQ** is thought to cause membrane disruption and facilitate the permeability of conventional antibiotics.

Before presenting compounds with novel mechanisms of action currently in (pre)clinical development, an overview of different therapeutic approaches to treat MAC lung disease using current anti-MAC antibiotics and repurposed anti-tuberculosis drugs is described.

## 5. Therapeutic Approaches for the Treatment of MAC Lung Disease

Various therapeutic approaches are currently being developed to treat MAC lung disease with improved safety and efficacy while limiting the risk of developing resistance. Clinical studies are being carried out to optimize GBT for improving tolerability and efficacy. New formulations, especially liposomal and inhaled therapies, are being developed to improve drug delivery to the lungs, enhance efficacy and reduce systemic side effects. In addition, the repositioning of certain well-known anti-tuberculosis drugs is also being investigated against MAC, particularly those with novel modes of action. Finally, host-directed therapies (HDT) are also a promising approach, but will not be discussed in this review. HDT consists of stimulating the macrophage response and preventing inflammation [47,48]. As discussed in the pathophysiology section, the immune system, and macrophages in particular, play an important role in the eradication of MAC. By targeting host processes, this approach complements therapy targeting the pathogen itself.

### 5.1. Optimized Combination Therapy

Contrary to MAC pulmonary infections, studies comparing combinations of **CLR** or **AZI** against disseminated MAC infections found no significant difference in efficacy. **CLR** is often used as a first-line treatment for MAC lung infections in France, but it is often poorly tolerated, particularly in terms of risk of hepatitis, metallic taste in the mouth, nausea or vomiting, and it interacts with many drugs via cytochrome P450. **AZI** has fewer side effects, especially less digestive toxicity and fewer drug interactions than **CLR**. So, on the assumption that **AZI**’s efficacy would be non-inferior to that of **CLR**, a phase III study (NCT03236987) is in progress to compare **RIF**-**EMB**-**CLR** tritherapy vs. **RIF**-**EMB**-**AZI.** This latter is also being studied in phase IV (NCT04921943) to determine whether a nebulized hypertonic saline can help improve symptoms and clearance of mycobacteria. Finally, this **RIF**-**EMB**-**AZI** tritherapy was also compared to **AZI**-**EMB** bitherapy for the treatment of non-cavitary MAC pulmonary disease in a phase III/II randomized pragmatic multicenter clinical study (NCT03672630).

Nowadays, a fixed-dose oral capsule including a combination of **RIB**, **CLR**, **CFZ**, is being studied in phase III (NCT04616924) in adult subjects with ND. Lanoix et al. have demonstrated synergistic in vivo activity between **CFZ** and **CLR** [49]. At month 4, **CLR**-**RIF**-**EMB** combinations were more effective than **CFZ**-**RIF**-**EMB** (2.0 ± 0.5 vs. 2.7 ± 0.2 mean lung CFU counts). However, when **CFZ** was added to **CLR**, efficacy improved as early as two months of treatment. Indeed, at month 4, the combination of **CLR**-**CFZ**-**RIF**-**EMB** is more effective than **CLR**-**RIF**-**EMB**.

A phase II study (NCT04287049) is currently underway, evaluating **AZI** in monotherapy for the first 14 days of MAC lung disease followed by treatment with GBT to assess its early bactericidal activity.

### 5.2. New Formulations: Liposomal and Inhaled Therapies

Liposomal and inhaled forms have been developed, particularly for **AMK** and **CFZ**.

- Aminosides: **AMK**

**Figure 6:**
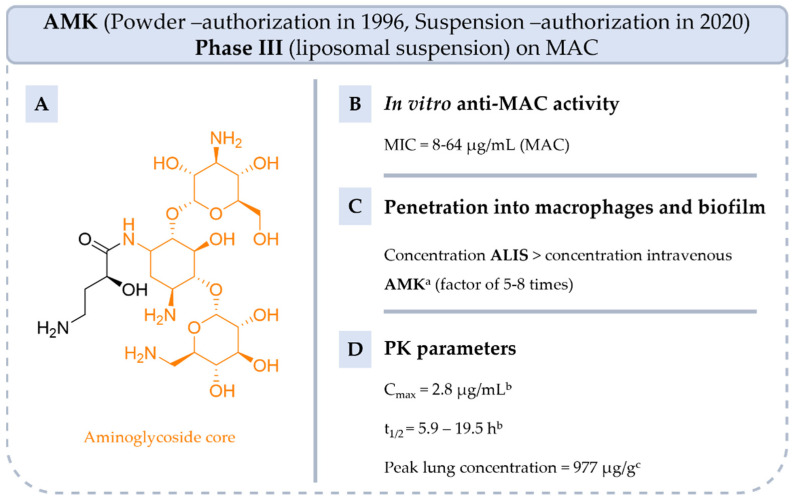
Structure of **AMK**, its main biological activities and PK parameters [38,50,51,54]. (**A**): Structure of **AMK** (aminoglycoside core in orange), (**B**): In vitro anti-MAC activity, (**C**): Penetration into macrophages and biofilm. ^a^ Male Han-Wistar rats treated with 40 mL at 53.4 mg/mL of **ALIS** or at 20 mg/mL of free **AMK**. The deposited dose determined at 2, 4 and 6 h post-dose. (**D**): PK parameters. ^b^ Parameters determined in young adult rhesus monkeys (*Macaca mulatta*) after intravenous administration of **AMK** at 20 mg/kg. ^c^ Parameters determined in male Han-Wistar rats after aerosol administration of **ALIS** or **AMK** at 96 mg/kg.

**AMK** is an aminoglycoside antibiotic introduced to the market as a powder for injectable solution in 1996 (Figure 6). **AMK** is a broad-spectrum antibiotic commonly used to treat bacterial infections caused by Gram-negative bacteria and certain types of Gram-positive bacteria, including those of the joints, urinary tract, skin, and soft tissues, especially when other antibiotics may not be effective due to resistance. It can also cure meningitis and multi-drug-resistant tuberculosis.

However, **AMK**, like other aminoglycosides, accumulates poorly in cells, potentially reducing its efficacy against intracellular infections and biofilm [50]. Intravenous injection of liposome-encapsulated **AMK** was more effective against disseminated MAC infections but not lung infections in mice [50]. Indeed, MAC pulmonary disease requires high antibiotic concentration in the lungs, but to limit toxicity, it is necessary to maintain low systemic levels. To address this, an inhaled liposomal formulation of **AMK** (**ALIS**) was developed [51,52,53,54].

**ALIS** is more effective in vitro than **AMK** alone with MAC isolates (minimal inhibitory concentration (MIC) = 8–64 µg/mL vs. MIC > 64 µg/mL) [52]. An in vivo study demonstrated that **ALIS** penetrates the biofilm and enhances the uptake of **AMK** by alveolar macrophages with a concentration 5–8 times higher than that of intravenous **AMK** at 2, 6 and 24 h post-dose in rats (e.g., C_macrophages_ = 4.0 µg/mL vs. 0.5 µg/mL at 24 h post-dose) [50]. Additionally, a higher sputum conversion (29.0% vs. 8.9%) and a lower systemic concentration in patients treated with 590 mg of **ALIS** compared with healthy adults who received an intravenous injection of **AMK** sulfate (area under the curve (AUC)_0–24h_ = 23.5 µg·h/mL, C_max_ = 2.8 µg/mL vs. AUC_0–24h_ = 154 µg·h/mL, C_max_ = 76 µg/mL) [53]. The average lung concentration is 10-fold higher with **ALIS** than with **AMK** [50].

Two Phase III clinical trials evaluating **ALIS** were completed in 2018 and 2023, respectively (NCT02344004 and NCT04677543). GBT was administered with or without **ALIS** in participants who were refractory to treatment (CONVERT study). The second trial studied **AZI** and **EMB** with or without **ALIS** (ARISE study). The first trial informs that for patients treated with **ALIS**, sputum culture conversion was achieved six months earlier than for patients receiving GBT alone. Adverse events such as renal toxicity and ototoxicity were relatively rare, but the combination of **ALIS** and **GBT** caused more respiratory side effects than GBT alone. Since 2020, **ALIS** has been on the market.

To date, the efficacy of **ALIS** is still being evaluated in combination with **AZI** and **EMB** in phase III trials (NCT04677569). In summary, the use of **ALIS** is therefore reserved for adults with MAC lung disease who have not responded to current treatments [19].

- Phenazines: **CFZ**

**Figure 7:**
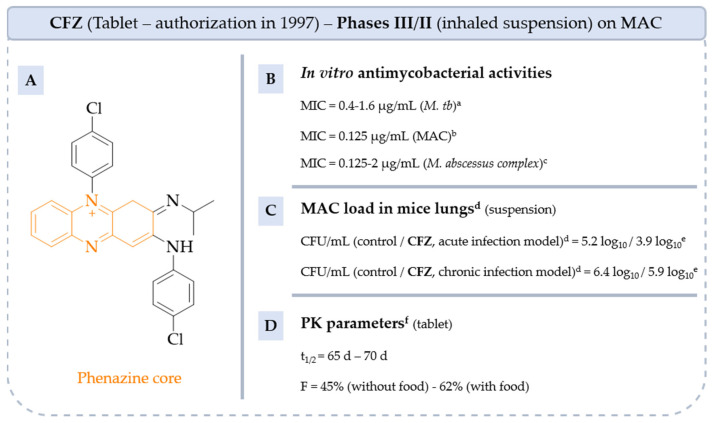
Structure of CFZ, its main biological activities and PK parameters [57,62,63]. (**A**): Structure of CFZ (phenazine core in orange), (**B**): In vitro antimycobacterial activities. ^a^ 10 strains of *M. tb* H37Rv. ^b^ *M. avium* ATCC 700898, *M. avium* B18101968. *M. intracellulare* DSM 43223, *M. chimaera* CIP 107892. ^c^ *M. abscessus* subsp. *abscessus* CIP 104536, B18104072, B15039863 and *M. abscessus* subsp. *massiliense* B12052284. (**C**): MAC load in mice lungs. ^d^ Beige mouse model with acute or chronic pulmonary infection of *M. avium* 2285R. ^e^ Reduction of *M. avium* 2285R load in lungs after intratracheal injection of CFZ inhalation suspension at 10 mg/kg. (**D**): PK parameters. ^f^ Parameters determined in humans after orally administration of 200 mg tablet taken with or without food.

**CFZ** is a phenazine antibiotic synthesized by Barry et al. in 1965 (Figure 7) [55]. Its marketing authorization was granted in 1997 in tablet form. **CFZ** is active against Mycobacterium species, including *M. leprae* and *M. tb*. It also shows some activity against Gram-positive bacteria, though it is not as widely used for general bacterial infections. Thus, **CFZ** is used in combination therapy to treat leprosy and has been used for several years off-label to treat multi-drug-resistant tuberculosis and MAC-infected patients intolerant of first-line treatment [1,5,56].

Due to its physicochemical (PC) properties (logP = 7.66, logD = 5.76 at pH 7.4), **CFZ** possesses low and variable oral absorption but a long half-life in humans and a concentration in the lungs, spleen, fat and plasma of mice of 800 mg/kg, 4000 mg/kg, 80 mg/kg and 3 mg/L, respectively [57,58].

To reduce systemic toxicity due to long-term oral absorption of **CFZ**, it is formulated as a microcrystalline suspension which can be delivered by inhalation, providing higher **CFZ** concentration in the lungs and a longer shelf life [59,60]. As an inhaled suspension form, the antimycobacterial activity of **CFZ** is preserved (MIC = 0.125 µg/mL) and the tolerated dose over 28 consecutive days in naive mice is 28 mg/kg [59]. Furthermore, in beige mouse models infected with acute and chronic NTM, the bacterial load is reduced. In the acute model, the load in the lungs when **CFZ** is administered orally or nasally is, respectively, 3.9 log_10_ and 3.4 log_10_ and more impressively 5.9 log_10_ and 2.4 log_10_ in the chronic infection model [59].

A study was conducted to determine the optimal dose of **CFZ** for the treatment of NTM diseases by oral administration (NCT05294146, phase II). In 2024, Jakko van Ingen pointed out that a dose of 100 mg per day administered orally in the treatment of NTM lung disease was considered effective, but that further studies were needed to confirm that a higher dose could reduce the duration of treatment [61]. Two clinical trials are currently underway to measure the efficacy and safety of **CFZ** alone as a tablet (NCT02968212, Phase II) in patients with MAC lung disease and as an inhaled suspension added to GBT (NCT06418711, Phase III).

### 5.3. Drug Repurposing

**Figure 8:**
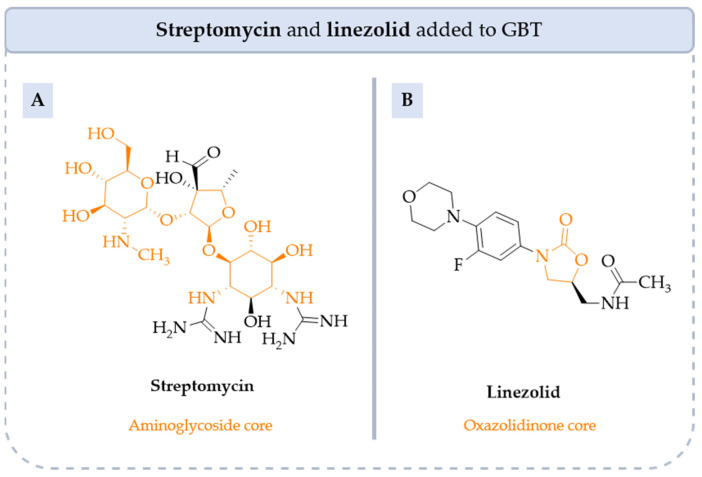
(**A**,**B**) Structures of **streptomycin** and **linezolid**.

**Figure 9:**
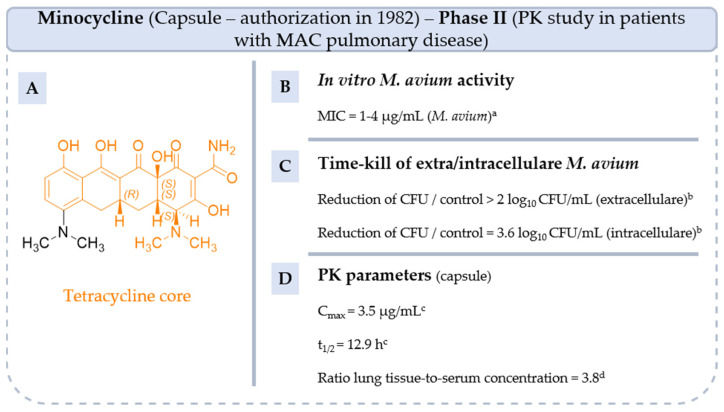
Structure of minocycline, its main biological activities and PK parameters [65,66,67]. (**A**): Structure of minocycline (tetracycline core in orange), (**B**): In vitro *M. avium* activity. ^a^ *M. avium* ATCC 700898. (**C**): Time-kill of extra/intracellular *M. avium*. ^b^ Maximal effect at 16-fold the MIC. (**D**): PK parameters. ^c^ Parameters determined in humans after oral administration of 200 mg of **minocycline**. ^d^ Parameter determined in male patients aged from 38 to 89 years after oral administration of 100 mg of **minocycline**.

**Figure 10:**
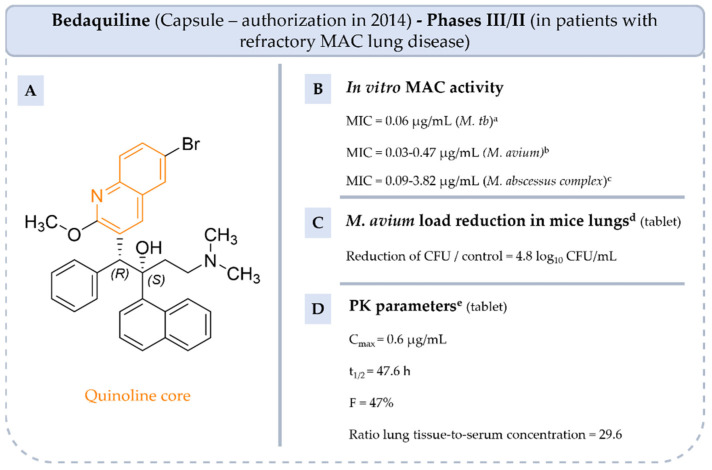
Structure of **BQ**, its main biological activities and PK parameters [40,68,69]. (**A**): Structure of **BQ** (quinoline core in orange), (**B**): In vitro antimycobacterial activities. ^a^ Drug-susceptible and multi-drug-resistant *M. tb* strains. ^b^ 9 clinical strains of *M. avium* ATCC 25291. ^c^ 13 clinical strains of *M. abscessus* subsp. *abscessus* and 9 clinical strains of *M. abscessus* subsp. *massiliense*. (**C**): *M. avium* load reduction in mice lungs. ^d^ BALB/c mice infected with *M. avium* ATCC 25291 and treated by oral gavage with 25 mg/kg of **BQ**. (**D**): PK parameters. ^e^ Parameters determined in BALB/c mice by oral gavage with 6.25 mg/kg of **BQ**.

**Streptomycin**, **linezolid**, **minocycline** and **BQ** are all antibiotics with known activity against *M. tb* and are currently being explored for their potential in treating MAC infections, especially in drug-resistant cases (Figure 8, Figure 9 and Figure 10). Whereas the use of both **streptomycin** and **linezolid** has significantly declined, **minocycline** and **BQ** are currently used in patients with MAC pulmonary disease (NCT05861258, NCT04630145).

**Streptomycin** is effective against various mycobacterial species. It is particularly active against RGM, but its effectiveness against SGM, like MAC, is more limited mainly because of its poor ability to penetrate membranes and effectively target these intracellular bacteria. Furthermore, **streptomycin** is rarely used due to its toxicity, particularly ototoxicity. Nevertheless, when combined with **AZI**, **RIB** or **EMB**, it can be beneficial in certain MAC treatment regimens, especially in cases involving resistance to other drugs.

**Linezolid** has demonstrated activity against MAC, particularly in cases involving resistance to first-line treatments or in immunocompromised patients. However, its use is limited by the risk of significant side effects, including myelosuppression and peripheral neuropathy, especially with long-term therapy.

**Minocycline** is a second-generation tetracycline first launched in the market in 1982 (Figure 9). Since its introduction, **minocycline** has been used to treat a variety of bacterial infections, including acne, respiratory infections, and sexually transmitted diseases, as well as for certain types of bacterial meningitis. Compared with first-generation tetracyclines, **minocycline** is able to penetrate tissues and cells more effectively, which enhances its ability to treat diseases caused by intracellular organisms [64]. **Minocycline** has been used in second-line treatment against *M. leprae*, particularly in drug-resistant cases and when no other medications are suitable. In addition, to its ability to inhibit the growth of MAC in vitro (MIC = 1–4 µg/mL against *M. avium* ATCC 700898), **minocycline** has suitable parameters to treat MAC pulmonary disease. Both extracellular and intracellular MAC are killed as concentrations of **minocycline** increase above the MIC, with maximum activity at 16-fold the MIC [65]. It is well-tolerated with an oral bioavailability close to 100%, a favorable penetration to the lung highlighted by a lung tissue-to-serum concentration ratio of 3.8 and a long half-life (t_1/2_ = 12.9 h) [65,66].

Furthermore, **minocycline** has shown additive or synergistic effects with anti-NTM antibiotics, making it an attractive option for inclusion in combined treatment regimens [65]. However, further clinical trials are needed to fully assess its efficacy and safety in MAC infections, especially in combination therapy with RIF which is known to affect the metabolism of tetracyclines. So, a clinical study has been carried out since 2023 to assess exposure to **minocycline** in MAC-pulmonary disease patients with and without concurrent use of **RIF** (NCT05861258, phase II).

**BQ** is a diaryl quinoline antibiotic, launched on the market in capsule form in 2014 to treat multi-drug-resistant tuberculosis (Figure 10). In 2018, it was registered with the World Health Organization (WHO) as part of the WHO’s List of Essential Medicines, promoting its use to treat multi-drug-resistant tuberculosis and extensively drug-resistant tuberculosis in patients for whom other treatment options were limited [68]. Indeed, its unique mechanism of action, which differs from conventional antibiotics, offers promise for treating resistant strains including MAC-resistant strains, especially when other drugs are ineffective. **BQ** is efficient against several NTM strains such as *M. avium* subsp. *avium* ATCC 25291, *M. chelonae* ATCC 14472, *M. abscessus* subsp. *abscessus* ATCC 19977 and 9–13 clinical isolates of these strains and 9 clinical strains of *M. abscessus* subsp. *massiliense* [69,70]. **BQ** showed good in vivo efficacy in BALB/c mice infected with MAC. The mean bacterial load was reduced by 4.8 log_10_ CFU in the mouse lung at week 4 of monotherapy compared to a control (administration of carboxymethylcellulose solution to mice) [69]. PK parameters of **BQ** were also determined, indicating a suitable C_max_ and t_1/2_, but moderate bioavailability due to high lipophilicity (logP = 7.5) [40]. However, this high lipophilicity favors its penetration into tissues and cells, enhancing its ability to eliminate intracellular organisms [71].

Currently, the efficacy of **BQ** is being evaluated and compared with **RIF** in combination with **CLR** and **EMB** in phase III/II study (NCT04630145) in adults with treatment-refractory MAC lung disease. However, the use of **BQ** is often limited by concerns about its potential side effects, including QT interval prolongation and hepatotoxicity, and its cost due to chemical synthesis. [72,73]. New anti-MAC compounds based on **BQ** scaffold are currently studied as, for example, the **sudapyridine** (**WX-081**) [68].

### 5.4. New Anti-MAC Compounds in (Pre)Clinical Development

In this section, we focus on anti-MAC compounds that have been the subject of both clinical and preclinical trials over the last six years. Compounds in preclinical trials were primarily selected based on their in vitro efficacy against MAC strains, specifically those with a MIC ≤ 8 µg/mL. The candidates examined in this review will be discussed according to the following points (i) chemical family and discovery, (ii) physicochemical (PC), pharmacokinetic (PK) and pharmacodynamic (PD) parameters, (iii) in vitro and in vivo biological properties and, iv) results from clinical trials.

Compounds in clinical trials for MAC include benzimidazole ureas such as **SPR719**/**SPR720** and benzoxaborole such as **epetraborole**. In addition, several families of compounds are being studied in the preclinical phase, including 5-phenylpyridine (**sudapyridine**), thiosemicarbazone (**SRI-286**), quinoline (**mefloquine**), **mavintramycin A** and indole-2-carboxamides (Figure 4).

#### 5.4.1. 5-Phenylpyridine: From **BQ** to **Sudapyridine**

**Figure 11:**
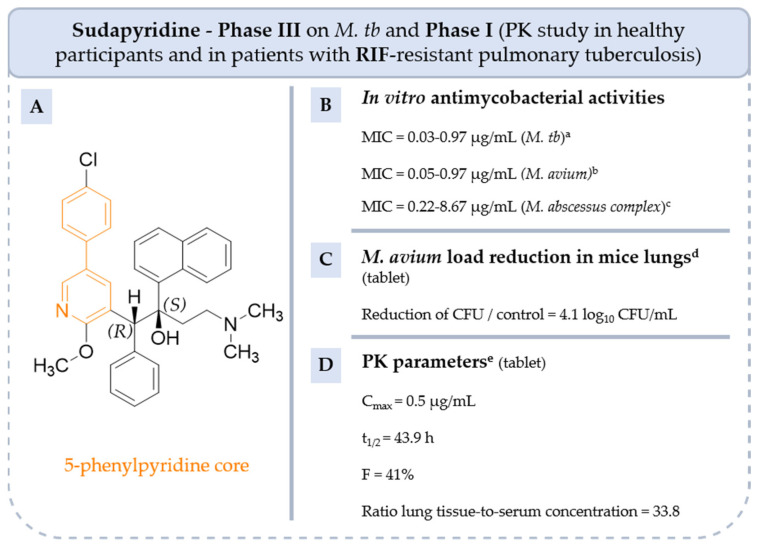
Structure of **sudapyridine**, its main biological activities and PK parameters [40,68,69]. (**A**): Structure of **sudapyridine** (5-phenylpyridine core in orange), (**B**): In vitro antimycobacterial activities. ^a^ Drug-susceptible and multi-drug-resistant *M. tb* strains. ^b^ 9 clinical strains of *M. avium* ATCC 25291. ^c^ 13 clinical strains of *M. abscessus* subsp. *abscessus* and 9 clinical strains of *M. abscessus* subsp. *massiliense*. (**C**): *M. avium* load reduction in mice lungs. ^d^ BALB/c mice infected with *M. avium* ATCC 25291 and treated by oral gavage with 25 mg/kg of **sudapyridine**. (**D**): PK parameters. ^e^ Parameters determined in BALB/c mice by oral gavage with 6.25 mg/kg of **sudapyridine**.

As previously described, **sudapyridine** is a 5-phenylpyridine, a simplified analog of **BQ,** and it was designed as an inhibitor of mycobacterial ATP synthase (Figure 11) [40]. **Sudapyridine** showed strong activity on a broad panel of mycobacteria such as sensitive and resistant strains of *M. tb* H37Rv (MIC = 0.03–0.97 µg/mL) and NTM strains such as *M. avium* ATCC subsp. *avium* 25291 (MIC = 0.05–0.97 µg/mL), *M. abscessus* subsp. *abscessus* ATCC 19977 and *M. abscessus* subsp. *massiliense* (MIC = 0.22–8.67 µg/mL) [40,68,69]. **Sudapyridine** also displayed good in vivo efficacy on BALB/c mice infected with MAC. The mean bacterial load was reduced by 4.1 log_10_ CFU/mL in the mouse lung at week 4 of monotherapy compared to a control (administration of carboxymethylcellulose solution to mice) [69]. Thus, the in vitro and in vivo antimycobacterial activities of **sudapyridine** were comparable to those of **BQ**. Yao, R et al. measured and compared the PK parameters of **BQ** and **sudapyridine** [40]. After exposure at a dose of 6.25 mg/kg *p.o.*, the ratios of lung tissue-to-serum concentration were slightly different (29.6 and 33.8 at 96 h, respectively, for **BQ** and **sudapyridine**). The bioavailability, half-time and maximum drug concentration of **BQ** and **sudapyridine** are similar (Figure 10 and Figure 11). However, peak time and AUC_0-inf_ of **sudapyridine** are higher than those of **BQ** by a factor of 1.4 and 1.9, respectively.

Studies showed that a high plasma concentration of the *N*-monodesmethyl metabolite of **BQ** can lead to significant QT prolongation whereas **sudapyridine** has a physiologically appropriate QT prolongation [74].

To date, **sudapyridine** tablets are being evaluated in combination with GBT and placebo in patients with **RIF**-resistant pulmonary tuberculosis and are being compared with **BQ** under the same conditions (phase III, NCT05824871). The safety and PK profile of **sudapyridine** has been, or is currently being, investigated in healthy volunteers (phase I, NCT06117514) and in patients with **RIF**-resistant pulmonary tuberculosis (phase I, NCT06701110 and NCT06701136).

#### 5.4.2. Benzimidazole Ureas: From Compound **1** to **Fobrepodacin** (Or **SPR720**)

**Figure 12:**
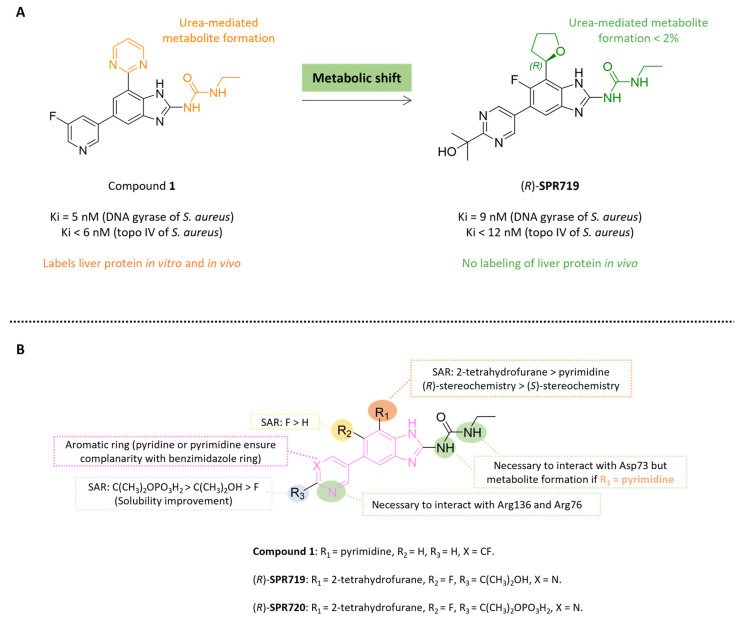
(**A**): Metabolic changes strategy to avoid reactive urea-mediated metabolite formation, (**B**): structure-activity SAR of benzimidazole ureas and key interactions with gyrase B from *S. aureus* in green (PDB ID: 4P8O) [75].

**Figure 13:**
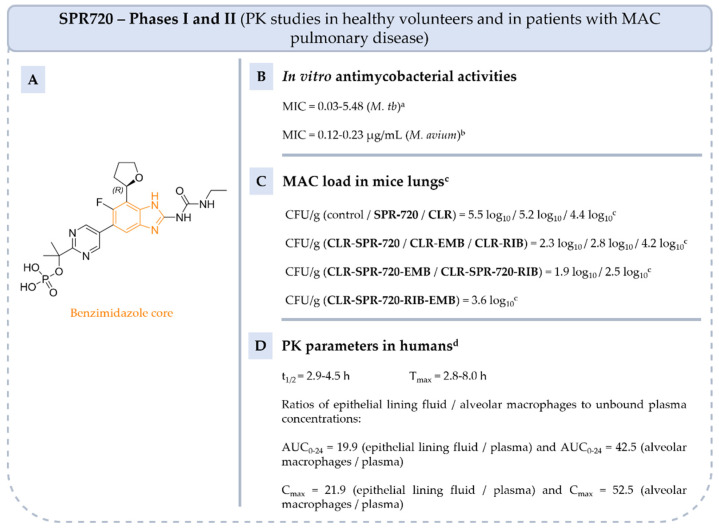
Structure of **SPR720**, its main biological activities and PK parameters [75,76,80,81]. (**A**): Structure of (*R*)-**SPR720** (benzimidazole core in orange), (**B**): In vitro antimycobacterial activities. ^a^ Drug-sensitive, multi-drug-resistant (resistant to **INH** and **RIF**) and extensively drug-resistant isolates of *M. tb*. ^b^ *M. avium* 103, Far and 3404.4 isolates. (**C**): MAC load in mice lungs. ^c^ C3HeB/FeJ mice infected with *M. avium* ATCC 700898 and treated by oral gavage with 30 mg/kg of **SPR720** alone or in combination with **CLR**, **EMB** and **RIB** every 24 h. (**D**): PK parameters in humans. ^d^ Parameters determined in healthy humans and treated by oral administration dose of 100 mg to 2000 mg of **SPR720**.

**SPR719** is a benzimidazole urea resulting from the fusion of benzene and an imi-dazole substituted by urea in position 2 (Figure 12 and Figure 13). Benzimidazoles ureas target the ATPase subunit (Gyr B subunit) of bacterial DNA gyrase and inhibit topoisomerase IV (ParE subunit), two enzymes involved in DNA replication and supercoiling in bacteria. [42].

In 2014, Grillot et al. identified compound **1** as a potent Gram-positive antibacterial agent through preclinical in vitro and in vivo studies (Figure 12). This compound exhibited very good in vitro bacterial activity against *S. aureus* ATCC 29213 (MIC = 0.016 µg/mL) and *S. pneumonia* ATCC 10015 (MIC ≤ 0.008 µg/mL) [75]. Its antibacterial activity was correlated to its strong inhibition of both gyrase B (inhibition constant (Ki) = 5 nM on *E. coli* gyrase B) and topoisomerase IV (Ki < 6 nM for *S. aureus* TopoIV) [75]. Crystal structure analysis of compound **1** in complex with *S. aureus* gyrase B (protein data bank identifiers (PDB ID): 4P8O) highlighted several key interactions: (i) a hydrogen bond between the C5-pyridine nitrogen of **1** and Arg136, (ii) a cation-π stacking interaction between the C5-pyridine ring of **1** and Arg76 and (iii) a bidentate hydrogen bond between the nitrogen’s urea and Asp73 [75] (Figure 12). In addition, the planarity between the pyrimidine and the benzimidazole rings is essential to maintain the activity towards gyrase B. Unfortunately, compound **1** presented a potential safety risk by covalently binding to liver proteins through the formation of a reactive metabolite, due to the presence of the urea group (Figure 12). Based on the above structural analysis, over 40 analogs were synthesized with urea mimics groups [75]. Since these structural modifications impair antibacterial activity, a metabolic change strategy was implemented, focusing on substitutions at the R_1_ position with 1-pyrazole or 2-tetrahydrofuran groups (Figure 12). These modifications ensure the planarity of the compounds by forming an intramolecular hydrogen bond between the R_1_-substituents and the benzimidazole, a preorganization ligand essential for targeting both bacterial gyrase B and topoisomerase IV. In addition, the substitution of benzimidazole core at C5 with a 2-(pyrimidin-2-yl)propan-2-ol moiety and at R_2_ position with fluorine to increase solubility and polar surface area (PSA = 125 Å^2^) led to **SPR719** [75]. These pharmacomodulations enabled a strategy of metabolic shift and reduced the urea-mediated metabolite formation that label liver proteins (<2%) [75].

During in vitro antibacterial evaluation, the activity of **SPR719** as the (*R*)-enantiomer was compared to that of its optical counterpart (*S*) and a racemic mixture. **SPR719** showed good in vitro activity against *S. aureus* ATCC 29213 (MIC = 0.032 µg/mL) and against *S. pneumonia* ATCC 10015 (MIC < 0.008 µg/mL), showing at least 15-fold and 2-fold greater potency compared to the *S*-enantiomer and the racemic mixture, respectively [75]. Subsequently, Locher et al. showed that **SPR719** had an in vitro activity against drug-sensitive and drug-resistant isolates of *M. tb* (MIC = 0.03–5.48 µg/mL) and a good affinity towards *M. tb* gyrase B (median inhibitory concentration (IC_50_ < 0.16 µg/mL and Ki < 0.39 µg/mL) [76]. By targeting gyrase B, **SPR719** is of particular interest because there is no risk of cross-resistance with fluoroquinolines that target DNA gyrase A.

Later, **SPR720**, the phosphate ester prodrug of **SPR719**, was developed to increase solubility and bioavailability. It has been used to treat MAC infections and has been recognized by the Food and Drug Administration (FDA) as a qualified product for infectious diseases, under fast-track and with orphan drug status. **SPR719** is active on NTM strains such as *M. abscessus*, *M. kansasii* and *M. avium* and multiple clinical strains (MIC = 0.002–4 µg/mL) [76,77,78]. In vivo studies have shown that **SPR720** is effective in mice models of MAC lung infection both as monotherapy and in combination with **CLR**, **RIB** and **EMB** [79]. While **SPR720** monotherapy reduces lung CFU compared to the control, its effect is less pronounced than that of **CLR** alone. However, when combined with **CLR**, **SPR720** leads to a greater reduction in MAC lung burden than the **CLR**-**EMB** or **CLR**-**RIB** combinations.

**SPR720** has been evaluated in three phase I clinical trials in healthy volunteers (NCT03796910, NCT05966688, NCT05955586) and in two phase II trials (NCT04553406 and NCT05496374) in patients with MAC pulmonary disease.

The first phase I clinical trial (NCT03796910), completed in 2019, showed that **SPR720** is well tolerated at daily doses of up to 1000 mg for up to 14 days and the main side effects reported were gastrointestinal and headache [80]. In order to determine the optimal oral dosing of **SPR720** in patients with NTM pulmonary disease, another phase I study (NCT05955586), now completed, was designed to determine the intrapulmonary PK of **SPR719** following the oral administration of multiple doses of **SPR720** (1000 mg, capsules). The last phase 1 study aims to evaluate the PK properties of **SPR720** when administered alone or in combination with **AZI** and **EMB** (NCT05966688). This trial was completed in February 2024, but no results have been published.

Finally, two phase II clinical trials (NCT04553406 and NCT05496374) in patients with NTM pulmonary disease to investigate the safety, tolerability, PK and efficacy of **SPR720** were completed in 2021 and 2024. The first study showed no adverse effects in patients who received 500 mg of **SPR720** orally every day for 28 days. The results of the second study have not yet been published.

#### 5.4.3. Benzoxaboroles: From **Tavaborole** to **Epetraborole** (**AN3365**)

Benzoxaboroles were first synthetized in 1957 by Torsell. They are pentacyclic boronic acid hemiesters that fused with benzene rings. The boron–carbon bond is more resistant to hydrolysis than the corresponding boronic acids and benzoxaboroles are generally more soluble in water [82,83].

**Figure 14:**
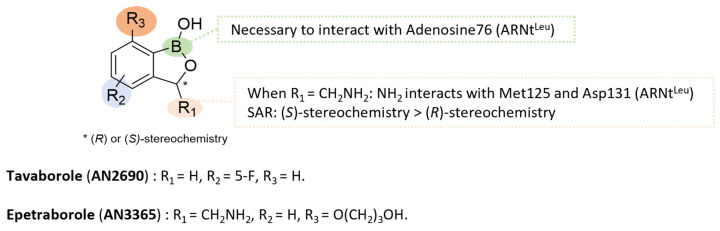
Structures of **tavaborole** and **epetraborole** and key interactions with *Thermus thermophilus* LeuRS in green (PDB ID: 2V0G) or with *M. abscessus* LeuRS editing domain in complex with **epetraborole**-AMP adduct in green and in beige (PDB ID: 7N12).

**Tavaborole** (**AN2690**) is the first compound of this class approved by the FDA in 2014 for the treatment of fungal infection, particularly onychomycosis [84]. **Tavaborole** inhibits leucyl-tRNA synthetase (LeuRS), an essential enzyme for the attachment of leucine to its corresponding tRNA, enabling accurate translation of the genetic code. Crystal structure analysis of the *Thermus thermophilus* LeuRS editing domain in complex with tRNA^Leu^-**tavaborole** adduct revealed an interaction between the boron atom of benzoxaborole and the 2′- and 3′-oxygen atoms of tRNA’s 3′-terminal adenosine 76 (PDB ID: 2V0G) (Figure 14) [84].

Later, Hernandez. et al. studied this class of drugs and its mechanism of action for Gram-negative infections. Modulation of the benzoxaborole core by aminomethyl substituent in R_1_ and *O*-propanol in R_3_, leading to **epetraborole** (or **AN3365**). The mechanism of action of **epetraborole** on NTM was confirmed by selecting mutanta in the gene encoding LeuRS (*leuS*, *MAB_1923c*) from *M. abscessus* Bamboo or *M. abscessus* ATCC 11977 [85,86]. The study of the crystal structure of the *M. abscessus* LeuRS editing domain in complex with **epetraborole**-AMP adduct highlighted key hydrogen bonds between the primary amine of **epetraborole** hydrogen and Met125 or Asp131 of *M. abscessus* LeuRS (PDB ID: 7N12) (Figure 14).

**Figure 15:**
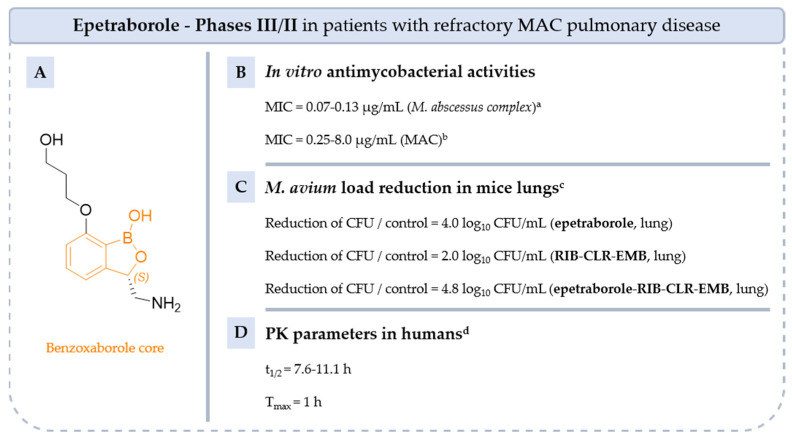
Structure of epetraborole, its main biological activities and PK parameters [86,87,88,89]. (**A**): Structure of (*S*)-epetraborole (benzoxaborole core in orange), (**B**): In vitro antimycobacterial activities. ^a^ Reference strains and clinical isolates of *M. abscessus complex*. ^b^ 51 isolates of MAC. (**C**): *M. avium* load reduction in mice lungs. ^c^ C57BL/6 mice infected with *M. avium* ATCC 700898 then treated by oral gavage with 200 mg/kg of **epetraborole** alone or in combination with **CLR/RIB/EMB** once daily. (**D**): PK parameters in humans. ^d^ Parameters determined in healthy adults given **epetraborole** tablets at dosages of 250–1000 mg every 24 h or 1000 mg every 48 h for up to 28 days.

**AN3365** was 281 times more active against *E. coli* LeuRS enzyme than its optical counterpart (*R*) and it was 0.6 times more potent than racemic mixture (IC_50_ = 0.31 µM vs. 87.2 µM vs. 0.54 µM) [75]. **Epetraborole** was active in vitro against *E. coli* ATCC 25922 and *P. aeruginosa* ATCC 27853 (MIC = 0.5–4 µg/mL) and different NTM strains such as *M. abscessus complex* and MAC (51 isolates: MIC = 0.07–8.0 µg/mL) (Figure 15) [86,87,88]. Moreover, **epetraborole** was assessed in vivo against 5 strains of MAC using a murine model of chronic infection [89]. It was administered both as monotherapy and in combination with GBT (**RIB**-**CLR**-**EMB**). In monotherapy, **epetraborole** demonstrated superior efficacy compared to GBT against *M. avium* ATCC 700898, and comparable efficacy against other strains. In monotherapy and at a dose of 200 mg/kg, **epetraborole** reduced the bacterial burden by 4.0 log_10_ CFU/mL on the reference *M. avium* ATCC 700898 strain after 28 days. Used in combination with GBT, **epetraborole** achieved a more pronounced reduction in lung CFU of 4.8 log_10_ compared with 2.0 log_10_ for the combination alone [89].

Oral administration of **epetraborole** to healthy volunteers at doses ranging from 250 mg to 1000 mg for 28 days was well tolerated, with no serious adverse events reported (phase I, NCT04892641) [90]. Phase 1b results showed a T_max_ of around 1 h post-dose and a t_1/2_ ranged from 7.6 to 11.1 h [91]. Since 2022, **epetraborole** has been the subject of a phase III/II trial designed to analyze the clinical responses of this drug in combination with an optimized background regimen (OBR) versus placebo + OBR in patients with refractory MAC lung disease (NCT05327803) [90].

#### 5.4.4. Thiosemicarbazones: From **Thiacetazone** to **SRI-286**

**Figure 16:**
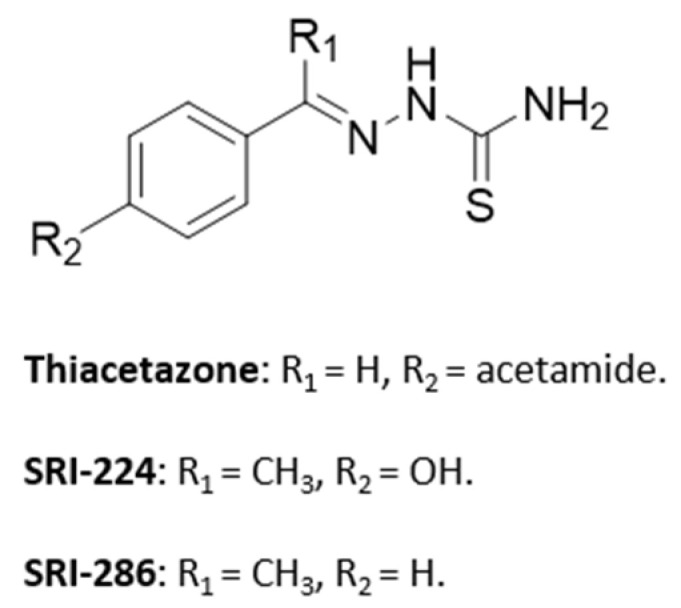
Structures of **thiacetazone**, **SRI-224** and **SRI-286**.

Thiosemicarbazones are a class of compounds belonging to the Schiff base family and are obtained by condensation of aldehyde or ketone with thiosemicarbazide. Among thiosemicarbazones, **thiacetazone** (or **amithiozone**) is an oral antibiotic which was used to treat tuberculosis during 1948 (Figure 16) [92]. **Thiacetazone** is a prodrug activated by S-oxidation of the thiocarbonyl moiety by monooxygenase EthA. The active form released inhibits InhA, a specific enzyme of FAS-II, involved in the biosynthesis of mycolic acids. [93,94,95]. In the 1950s, it fell into disuse due to its toxicity (dermatological toxicity) and the discovery of more active drugs such as **INH**. However, thiosemicarbazones continued to attract interest as antimycobacterial agents, thanks to their low cost of synthesis [95,96,97]. Moreover, in 1990, the anti-NTM activity of **thiacetazone** was reported. This compound was generally more active against a panel of clinical *M. avium* strains than against *M. tb* H37Rv strains (MIC = 0.02–0.15 µg/mL vs. 0.08–1.2 µg/mL) [98].

**Figure 17:**
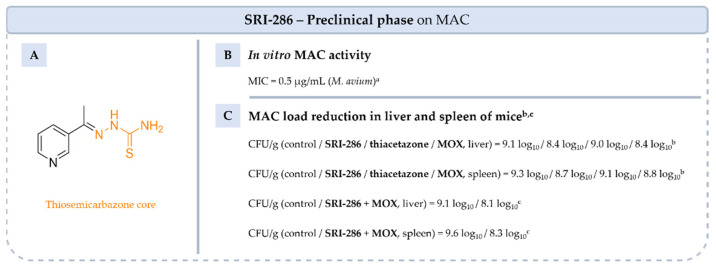
Structure of **SRI-286**, its main biological activities and PK parameters [97]. (**A**): Structure of **SRI-286** (thiosemicarbazone core in orange), (**B**): In vitro MAC activity. ^a^ MAC strains 100, 101, 108, 109 and 116. (**C**): MAC load in liver and spleen of mice. ^b^ C57BL/6J-Lyst^bg-J^/Lyst^bg-J^ female mice infected with MAC 101 and treated with **SRI-286** or **thiacetazone** at 40 mg/kg or **MOX** at 100 mg/kg by oral gavage. ^c^ C57BL/6J-Lyst^bg-J^/Lyst^bg-J^ female mice infected with MAC 101 and treated with **SRI-286** and **MOX** at 40 mg/kg and 100 mg/kg, respectively, by oral gavage.

From 2000 to 2015, **thiacetazone** was studied in phase II clinical trials in association with **CLR**, **streptomycin**, **EMB** and **RIF** in patients with MAC pulmonary disease (NCT00004689). Although **thiacetazone** has shown satisfactory results in clinical trials, Bermudez et al. identified numerous thiosemicarbazone analogs such as **SRI-224** and **SRI-286** with 2- to 256-fold greater activity against various MAC strains. [97]. Thereafter, in vivo studies demonstrated that **SRI-286** significantly inhibited bacterial growth in the liver and spleen (Figure 17).

In mice infected with MAC 101, treatment with **SRI-286** at a dose of 40 mg/kg/day further inhibited mycobacterial growth in the liver and spleen compared to untreated mice [97]. **SRI-286** is slightly more effective than **thiacetazone** and as active as **moxifloxacin** (**MOX**). Additionally, the mean number of CFU was reduced in liver and in the spleen, when **SRI-286** is in association with **MOX** compared to monotherapy (Figure 17).

#### 5.4.5. **Mefloquine**

**Figure 18:**
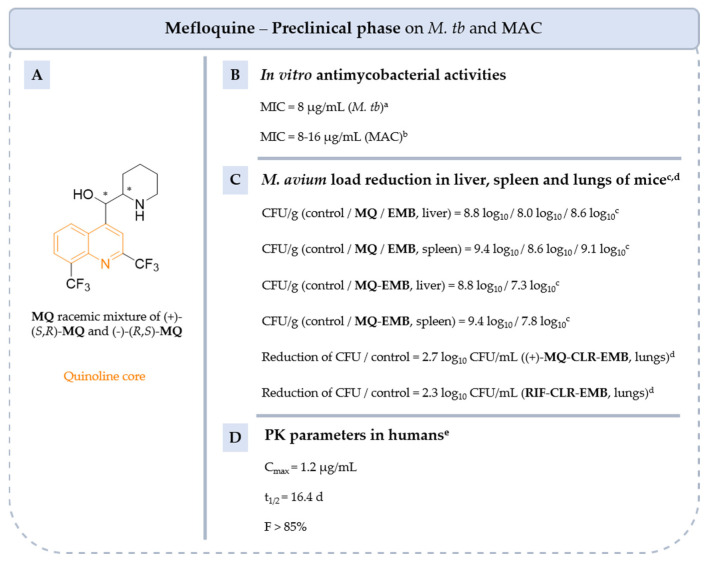
Structure of **MQ**, its main biological activities and PK parameters [100,101]. (**A**): Structure of **MQ** (quinoline core in orange), (**B**): In vitro antimycobacterial activities. ^a^ *M. tb* H37Rv. ^b^ MAC 101, 511 to 513, JJL and JWT. (**C**): *M. avium* load reduction in liver, spleen and lungs of mice. ^c^ Female C57BL/6 *bg^+^ bg^+^* mice infected with MAC 101 and treated with 40 mg/kg/d of **MQ** and with 100 mg/kg/d of **EMB** by daily gavage for 28 days. ^d^ Female BALB/c mice infected with *M. avium* ATCC 700898 and treated with (+)-**MQ**-**CLR**-**EMB** or **RIF**-**CLR**-**EMB** combinations at 8 mg/mL (**MQ**), 2 mg/mL (**RIF**) and 20 mg/mL (**CLR** and **EMB**). Antibiotics are administered orally by gavage 5 days per week for 3 months. (**D**): PK parameters in humans. ^e^ Parameters determined in healthy humans and treated by oral administration dose of 750 mg of racemic **MQ**.

**MQ** contains a quinoline pharmacophore, i.e., a benzene fused to a pyridine (Figure 18). **MQ** was first developed in 1970 and is used commercially as an *erythro* racemic mixture for its antimalarial activity. The antibacterial mechanism of action of **MQ** is not fully understood; however, evidence suggests that it targets ATP synthase, an essential enzyme in the cellular energy production pathway like **BQ** [43]. Recent studies suggest that **MQ** disrupts membrane integrity and, therefore, increases permeability, enhancing the efficacy of conventional antibiotics [44,45,46]. **MQ** may also inhibit efflux pumps [45].

In 2014, a study showed that **MQ** is active against *M. tb* H37Rv and on THP-1 macrophages infected with *M. tb* [99]. Additionally, **MQ** showed a synergistic effect with first-line antituberculosis drugs such as **INH** and **pyrazinamide** against *M. tb* H37Rv [44]. **MQ** also demonstrated in vitro efficacy against different strains of MAC, including **CLR-resistant** strains [100]. Due to these encouraging results, the in vivo activity of **MQ** was evaluated in beige mice infected with MAC 101 and in BALB/c mice infected with the reference strain *M. avium* ATCC 700898 [100,101]. The first study demonstrated that a dose of 40 mg/kg/d administered alone or in combination with **EMB**, decreased the number of CFU by a factor of 1.1 log_10_ in the liver and 1.2 log_10_ in the spleen [100]. The second study reported that administering (+)-**MQ** at 40 mg/kg in combination with **CLR** and **EMB**, 5 days per week over a 3-month period, was as effective as the currently used combination of **RIF**, **CLR** and **EMB** [101]. When combined with **CLR** and **EMB**, the (+)-*erythro* enantiomer showed better activity, reducing the number of CFU by a factor of 1.1 and 1.2 compared with the racemic mixture and (−)-**MQ** enantiomer [101]. These results confirm those previously reported by Bermudez et al. [102]. PK parameters of racemic **MQ** were also determined in healthy volunteers after a single oral dose of 750 mg, indicating a suitable C_max_, a very long t_1/2_ and a good bioavailability improved to around 40% with food [103].

#### 5.4.6. **Mavintramycin A**

**Figure 19:**
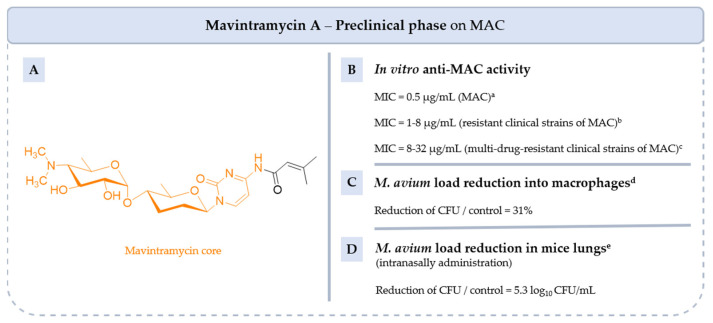
Structure of **mavintramycin A**, its main biological activities and PK parameters [104]. (**A**): Structure of **mavintramycin A** (mavintramycin core in orange), (**B**): In vitro anti-MAC activity. ^a^ *M. avium* JCM15430, *M. intracellulare* JCM6384, *M. avium* ATCC 700898. ^b^ 40 clinical strains of *M. avium* resistant to **CLR**, **RIF** or **EMB**. ^c^ Multi-drug-resistant strains of MAC. (**C**): *M. avium* load reduction into macrophages. ^d^ THP-1 macrophages infected with *M. avium* and treated with 10 µg/mL of **mavintramycin A** for 4 days. (**D**): *M. avium* load reduction in mice lungs. ^e^ Female BALB/c mice infected with *M. avium* JCM15430 and treated with 10 mg/kg/d of **mavintramycin A** for 7 days by intranasally administration.

Mavintramycins are compounds from the marine strain *Streptomyces* sp. OPMA40551 containing cytosine, amosamine and amicetose moieties (Figure 19). Among several mavintramycins tested by Hosoda et al., **mavintramycin A** was the most active in vitro on *M. avium* JCM15430, *M. intracellulare* JCM6384 and *M. avium* ATCC 700898 [104]. The latter also showed very good activity on 40 clinical strains of *M. avium* resistant to **CLR**, **RIF** or **EMB** and on 18/20 multi-drug-resistant strains. Moreover, THP-1 macrophages infected with *M. avium* and treated with **mavintramycin A** for 4 days resulted in a 31% reduction in the number of *M. avium* colonies compared to the control.

The bactericidal or bacteriostatic effect of the compound was also studied. **Mavintramycin A** inhibited 50% of *M. avium* at a MIC dose of 3.12 µg/mL [104]. At 10 times the MIC, bacterial growth was completely inhibited, indicating that the compound has a bactericidal effect against *M. avium*. Additionally, at the compound’s MIC, and in the presence of **CLR** at 10 times its MIC, bacterial growth was halted within 2 days.

The investigation of synergistic action of **mavintramycin A** with **CLR** and **RIF** indicated an additive effect, while an antagonistic interaction was observed with **EMB**. To further assess its potential, the efficacy of **mavintramycin A** was evaluated in *M. avium*-infected mice. The compound was administered intranasally at a dose of 10 mg/kg per day for 7 days. Treatment resulted in a 5.3 log_10_ CFU/mL reduction in the lungs compared to the control, demonstrating strong in vivo activity. Mechanistic studies indicated that **mavintramycin A** inhibits protein synthesis by binding to 23S rRNA.

#### 5.4.7. Indole-2-Carboxamides

The MmpL3 transporter is responsible for the translocation of mycolic acids in the form of trehalose monomycolate from their production site in the cytoplasm to the cell envelope. Blockage of the MmpL3 transporter leads to weakness of the bacterial cell wall and impacts on the viability and virulence of mycobacteria. The MmpL3 transporter is, therefore, a highly attractive target for combating MAC strains.

**Figure 20:**
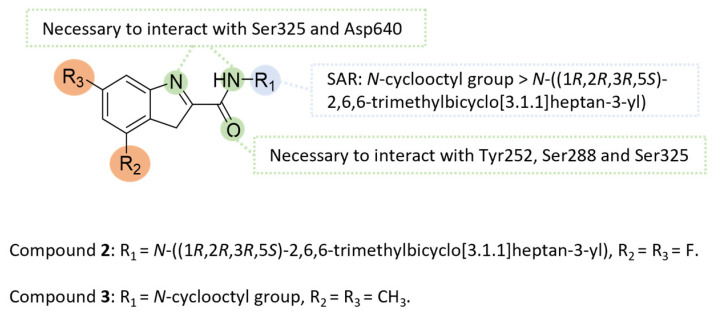
SAR of indole-2-carboxamides and key interactions with the MmpL3 transporter from *M. tb* in green (PDB: 7NVH) [106].

In the 2010s, the indole-2-carboxamide scaffold was extensively investigated [105,106]. Among these compounds, a lead molecule (lead 2) was subjected to molecular docking studies with the MmpL3 transporter from *M. tb* using its crystal structure (PDB: 7NVH) (Figure 20) [106]. The analysis revealed several key interactions, including hydrogen bonds between indole nitrogen and Ser325, and carboxamide nitrogen and Asp640, and carboxamide carbonyl oxygen and Tyr252, Ser288 and Ser325 [106].

**Figure 21:**
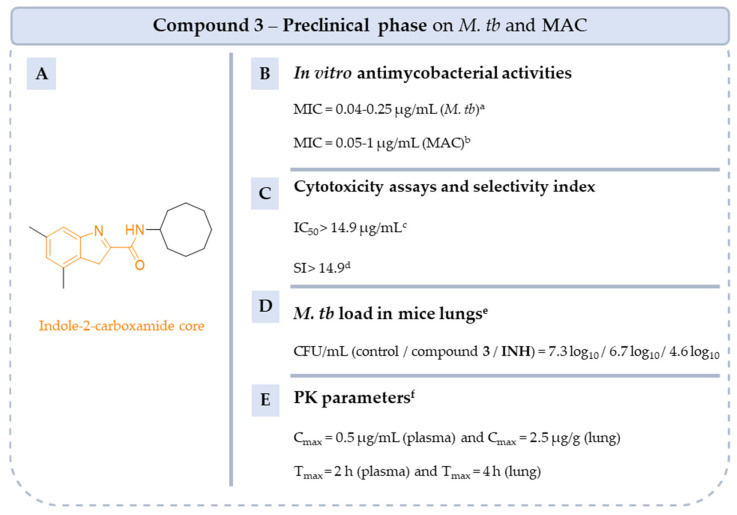
Structure of compound **3**, its main biological activities and PK parameters [75,76,80]. (**A**): Structure of compound **3** (indole-2-carboxamide core in orange), (**B**): In vitro antimycobacterial activities. ^a^ *M. tb* H37Rv and indoleamide-resistant *M. tb* strain (IAR2 strain). ^b^ *M. avium* 104 and *M. intracellulare* 1956. (**C**): Cytotoxicity assays and selectivity index. ^c^ IC_50_ determined against THP-1 cells. ^d^ SI (IC_50_/MIC) on *M. avium* 104 and *M. intracellulare* 1956. (**D**): *M. tb* load reduction in mice lungs. ^e^ Female BALB/c were aerosol-infected with *M. tb* H37Rv. Mice were treated with 300 mg/kg of compound **3** or with 10 mg/kg of **INH** by oral gavage 5 days per week for 28 days. (**E**) PK parameters. ^f^ Parameters determined in female BALB/c and treated by oral administration dose of 100 mg of compound **3**.

Additionally, numerous compound **2** analogs were synthesized and evaluated in vitro on *M. tb* [105,106]. Compound **3** showed very good activity on the reference strain of *M. tb* H37Rv and on the drug-resistant strain of *M. tb* (Ser to the missense mutation at position 288 of *Rv0206c* gene) (Figure 21) [105]. Th =e study of the PC parameters showed that compound **3** complies with the Lipinski rules (just the clogP of 5.6 is above 5) indicating satisfactory oral absorption and permeability. In vivo PK parameters in mice confirm good oral bioavailability. Compound **3** is also active in vivo on *M. tb*. Female BALB/c mice were infected by aerosol with *M. tb* H37Rv and compound **3** was orally administered daily at doses of 33, 100 and 300 mg/kg. After 4 weeks of treatment, the number of CFU counts in the lungs was reduced by 0.6 log_10_ compared to untreated mice, although the reduction was less than with **INH** treatment (reduction of 2.7 log_10_) [105]. Based on these promising initial results, indole-2-carboxamides were studied on NTM. In 2017, Franz et al. evaluated indole-2-carboxamide **3** on THP-1 cells. Compound **3** is not cytotoxic above 14.9 µg/mL against THP-1 cells. Thus, the selected indole-2-carboxamide **3** showed a good selectivity index against *M. avium* 104 and *M. intracellulare* 1956 (SI > 14.9) [107]. These results highlight the need to continue preclinical studies with a view to their application as drugs.

All molecules cited in this review illustrate the diversity of compound families and their modes of action (Table 2). Although all these classes hold promise for the treatment of MAC infections, they were originally developed to treat tuberculosis, making them non-specific drugs against NTM. In addition, the number of new anti-MAC agents is still too low to prevent the emergence of this NTM. It is therefore important to maintain efforts to develop drugs against MAC and increase the number of clinical trials.

## 6. Conclusions

MAC is the most prevalent NTM worldwide, accounting for approximately 80% of NTM-related pulmonary infections. Current treatment relies on GBT, typically involving a combination of three antibiotics, including a macrolide. However, treatment is prolonged, often poorly tolerated, and only moderately effective. The therapeutic challenge is intensified by MAC’s intrinsic resistance mechanisms, in particular due to its thick, hydrophobic cell wall and complex antibiotic resistance pathways. To overcome these obstacles, research has focused on optimizing therapeutic regimens, repurposing existing drugs, and identifying novel anti-MAC agents. These strategies have led to the study of several families of compound targeting various bacterial processes, from membrane integrity to protein synthesis. Additionally, synergistic drug interactions and advanced formulations have shown promise in enhancing antimycobacterial efficacy and improving pharmacokinetic and toxicity profiles. These two approaches make it possible to obtain more effective molecules while shortening development time compared with the study of new agents, which could make them priority strategies. This review highlights several antibiotic families under investigation, underscoring the therapeutic potential of these emerging strategies to improve treatment outcomes against MAC pulmonary infections.

Currently prescribed drugs or new therapeutic options to treat NTM infections are developed from treatments aimed at eradicating *M. tb*. In the future, it will be essential to develop new drugs specifically designed to combat NTMs, and MAC in particular, in order to improve the outcome of MAC lung infections. Whether it is a question of modifying galenic formulations, repositioning certain molecules or developing new ones, we need to focus on clinical evaluation, first in vivo and then in randomized studies. The latter are still too few in number, even if they are on the increase. It should not be forgotten that the majority of international recommendations are still based on expert recommendations. This will also help in discussions with administrative authorities to enable easier patient access to new molecules, even before these treatments are marketed.

## Figures and Tables

**Table 1:** Treatment of MAC pulmonary disease according to European and North American recommendations.

Type of Disease	Regimen	Recommendations	
		European	North American
ND	GBT: **CLR**/**AZI** **RIF** **EMB**	**3 times weekly or daily**	
		1000 mg/250 mg 10 mg/kg 15–20 mg/kg	1000 mg/500 mg 600 mg 25 mg/kg
CD	GBT: **CLR**/**AZI** **RIF** * * **EMB** And * * **AMK** IV	**3 times weekly or daily**	
		1000 mg/250 mg 10 mg/kg Or **RIB**: 150–300 mg 15 mg/kg And * * 10 mg/kg	1000 mg/250–500 mg 450–600 mg * * 15 mg/kg And * * 15 mg/kg
RD	**(daily)** GBT and **ALIS** or **AMK** IV (or **streptomycin**)		

**Table 2:** Anti-MAC molecules cited in the review, their mode of action and specifically development against MAC (x means that the compounds are not specifically developed against MAC).

Molecules	Mode of Action	Develop Specifically Against MAC
Fobrepodacin	Inhibition of DNA replication	-
Rifampicin	Inhibition of transcription	-
Rifamycin		-
Clarithromycin	Inhibition of protein synthesis	-
Azithromycin		-
Streptomycin		-
Linezolid		-
Amikacin		-
Minocycline		-
Epetraborole		x
Mavintramycin A		x
SRI-286	Inhibition of mycolic acids biosynthesis	-
Ethambutol	Inhibition of arabinogalactan biosynthesis	-
Indole-2-carboxamides	Inhibition of MmpL3 transporter	-
Clofazimine	Inhibition of the electron transport chain (NADH-quinone oxidoreductase II)	-
Bedaquiline	Inhibition of the electron transport chain (ATP synthase)	-
Sudapyridine		-
Mefloquine	Inhibition of ATP synthase, efflux pumps and disruption of cell membrane integrity	-

## Data Availability

No new data were created or analyzed in this study. Data sharing is not applicable.

## Abbreviations

ALIS	amikacin liposome inhalation suspension
AMK	amikacin
ATS	American thoracic society
AUC	area under the curve
AZI	azithromycin
BQ	bedaquiline
CD	cavitary disease
CF	cystic fibrosis
CFU	colony-forming unit
CFZ	clofazimine
CLR	clarithromycin
COPD	chronic obstructive pulmonary disease
EMB	ethambutol
FDA	Food and Drug Administration
GBT	guidelines-based therapy
HDT	host-directed therapies
IC_50_	median inhibitory concentration
IDSA	Infectious Diseases Society of America
INH	isoniazid
Ki	inhibition constant
MAC	*Mycobacterium avium* complex
MIC	minimal inhibitory concentration
MOX	moxifloxacin
*M. tb*	*Mycobacterium tuberculosis*
MQ	mefloquine
ND	nodular bronchiectasis disease
NTM	non-tuberculous mycobacteria
PC	physicochemical
PD	pharmacodynamical
PDB ID	protein data bank identifiers
PK	pharmacokinetic
PSA	polar surface area
RD	refractory disease
RIB	rifabutin
RIF	rifampicin
RGM	rapidly growing mycobacteria
SAR	structure-activity relationships
SGM	slowly growing mycobacteria
SI	selectivity index
WHO	World Health Organization
